# New York Academy of Medicine—Section on Orthopedic Surgery

**Published:** 1892-05

**Authors:** 


### NEW YORK ACADEMY OF MEDICINE.—SECTION ON ORTHOPEDIC SURGERY.

*Stated Meeting, March 18, 1892.*

HENRY LING TAYLOR, M. D., Chairman.

### ASYMMETRY OF THE EXTREMITIES.

Dr. L. W. HUBBARD presented two sisters exhibiting this condition. One child had one and a half inches shortening of the left lower extremity, and about two and a half inches shortening in the left upper extremity, which was about equally divided between the arm, forearm and hand. There was also slight shortening of the left ramus of the jaw. Her younger sister also exhibited about the same amount of shortening of the left upper and lower extremities. The muscles were developed in both cases. Their parents were healthy Germans, and there was no history of a similar deformity in other members of the family. An attempt had been made to explain this asymmetry on the theory that there is an unequal development of the cerebrum on the two sides.

Dr. A. B. JUDSON had seen a counterpart of these cases in a girl of eleven years, in whom the right ear and eye, as well as the right upper and lower limbs, were congenitally smaller than the left. He suggested wearing an ischiatic crutch on the larger side, and a high sole on the smaller side during the periods of rapid growth. He thought that hip cases treated in this way owed the disparity in length of the limbs, which is found in the tibia, as well as in the femur, partly to the disease of one, and the over-use of the other. Advantage should be taken of this fact in the treatment of these cases of congenital asymmetry.

Dr. R. H. Sayre said that many writers had denied that want of symmetry in the lower extremities is a cause of true lateral curvature, and that the occasional association of the two conditions is a mere coincidence. Personally, however, he believed that if the children first presented were allowed to go on to puberty without the employment of measures to equalize the limbs, they would certainly develop true lateral curvature. In one of the cases the lack of development did not seem to him to be entirely confined to one-half of the body, as the left side of the face appeared larger than the right, although the extremities were smaller on the left side than on the right. On this account, he did not think the theory that this asymmetry was due to unequal development of the two halves of the cerebrum, could be correct.

He agreed with the previous speaker that much of the atrophy following hip disease was due to lack of use, and he therefore heartily endorsed his suggestions as to treatment.

Dr. A. M. Phelps said that his experience had led him to believe that the shortening of the limb in hip disease is never due to anything but bone destruction, and that the employment of the treatment suggested would effect no change in the length of the limbs, although it might increase their circumference.

Dr. R. H. Sayre said that after cases of club-foot have improved sufficiently to enable them to use their feet, it is noticed that there is not only an increase in the bulk of the feet, but also in the length of the bones. It had also been observed in colleges where careful records are kept of the physical condition of the students, that those who exercise regularly in the gymnasium not only have larger muscles, but are taller than those who do not avail themselves of this opportunity for physical training.

### RESULTS IN CASES OF HIP DISEASE TREATED BY THE PORTABLE TRACTION SPLINT WITHOUT COMPLETE IMMOBILIZATION EXCEPT DURING THE INFLAMMATORY STAGE; WITH ILLUSTRATIVE CASES AND PHOTOGRAPHS OF CASES.

Dr. Lewis A. Sayre read a paper with the above title. He held that absolute immobilization of the diseased joint during the entire period of treatment, as advocated by a number of writers in the past few years, was not always essential to complete recovery, and he presented detailed histories of seven cases in support of this view. The diagnosis in all these cases had been confirmed by other surgeons of recognized ability, who had seen them in consultation. Photographs of five of these patients were exhibited, which showed absolutely normal mobility of the joint, the photographs being taken with both legs straight, and the patient in the standing position, and also with the foot of the diseased side on top of a chair, and again, with the patient sitting with the foot of the diseased side on the knee of the opposite side, and the knee of the diseased side dropped so as to make the leg parallel with the floor. One patient was present who could do all motions equally well with either hip, and another, who was shown as a *good* but not as a perfect cure, who could put either foot on top a chair in front of him, and who could cross his legs, but who was unable to put the foot on the diseased side in his lap, as could all the other patients whose histories were reported in full.

Dr. Sayre then gave the following statistics of 407 cases of morbus coxarius, which he had treated between the years 1859 and 1889, exclusive of exsections :

First stage, 118 ; second stage, 119 ; third stage, 82 ; not mentioned, 88. Total number of cases, 407.

RESULTS.

Cured, motion perfect, 71 ; cured, motion good, 142 ; cured, motion limited, 83 ; cured, anchylosed, 5 ; unknown, 78 ; under treatment, 14 ; abandoned, 3; discharged, 2. Died of exhaustion, 2 ; died of phthisis, 1; died of pneumonia, 1; died of tubercular meningitis, 5. Total deaths, 9. Total number of cases, 407.

Cases in which the author knew the result, and also the kind of splint worn between 1859 and 1889, excluding cases under treatment. Cures with perfect motion, long splint, 19 or 21.59 per cent.; short splint 54 or 28.12 per cent. Cures with good motion, long splint, 34 or 38.63 per cent.; short splint, 86 or 44.79 per cent. Cures with limited motion, long splint, 29 or 32.95 per cent.; short splint, 49 or 25.52 per cent. Cures with anchylosis, long splint, 3 or 3.40 per cent.; short splint, 1 or 0.52 per cent. Deaths, long splint, 3 or 1.56 per cent.; short splint, 2 or 1.04 per cent. Treated with long splint, 88. Treated with short splint, 192. Total, 280.

The plan of treatment pursued in these cases had been rest in bed, usually with a blister behind the trochanter when the case was seen in the early stages, combined with traction in the line of the deformity with such weight as gave the greatest relief, while the body and sound limb were secured to a long splint passing from the axilla to the foot. In certain cases, lateral traction was also added. This was first used by Dr. Sayre in 1868. When the acuteness of the joint spasm had subsided, and the deformity had been overcome, the line of traction having been gradually changed until the limbs were parallel, the child was allowed to get up with the short splint and crutches in some cases, and in other cases with the long splint, either with or without crutches, according to circumstances. The author did not enter into the details of application, as these had previously been fully described. Cases involving both joints had been treated in the wire cuirass, as far as possible, in order to allow of driving in the sun and air. The limbs were occasionally removed from the cuirass, one at a time, for the purpose of making slight motion of the joint, inside the degree of causing pain. Traction was considered as one of the requisites of treatment, as the author had seen cases go on to extensive suppuration with entire destruction of the acetabulum, from reflex pressure, in spite of constant fixation by plaster of Paris for two years without traction. He had also seen a case of anchylosis of all the joints of the trunk and lower extremities in a case that was kept constantly in a cuirass for nine months without motion. The anchylosis in this case was not accompanied by any fever or pain, so that the supposition that a rheumatic diathesis was responsible for this condition, was untenable.

Cases of exsection had not been included in the table of statistics, as they had been published separately, and most of them had been in such advanced stages when first seen as to preclude the possibility of mechanical treatment.

### DISCUSSION.

Dr. Judson agreed with the writer of the paper that traction does not secure complete immobilization, but rather fixation, or a fractional and sufficient degree of immobilization. Fixation thus produced, relieves pain and hastens recovery, but does not prevent the correction of deformity, which is brought about conveniently and surely as soon as the patient, wearing the hip splint or the ischiatic crutch, is taught to observe habitually the natural rhythm of walking. Adduction and flexion are thus reduced, because the limb reaches outward and downward in abduction ; and extension, in order to do its share of the work of progression which is thrown upon it by the footsteps, is equalized. He had been pleased to find that not only is deformity reduced, but also the range of motion is increased in the joint when the limb is summoned in this way to do, as far as it can, its half of the work of locomotion.

Dr. Phelps said that while listening to the paper, he had been impressed with the striking difference between the statistics presented by the author and those published a few years ago by Shaffer and Lovett, notwithstanding that all these gentlemen used the same plan of treatment. In thirty-nine cases reported by the last two gentlemen named, nineteen had anchylosis, and seven were in a condition almost equivalent to anchylosis. The author of the paper which had just been presented, deserved to be congratulated on the large number of magnificent cures which he had obtained. The speaker admitted that he had become somewhat prejudiced against the long traction splint, partly as a result of experience, and partly because of the publication of the statistics which he had just quoted. Where anchylosis had occurred, he believed it was due to trauma which had been produced by allowing the patient to walk upon the apparatus, or on account of a joint in the splint which allowed free motion, or because traction had not been made in the axis of the neck. He considered that the introduction of the long traction splint marked a distinct advance in orthopedic surgery, but he thought still further advance would follow attention to the points just mentioned, and it was on this account that he had adopted the plan of complete immobilization. The long traction splint was born of a fear of anchylosis, and a desire that the patient should have exercise ; yet, in his own experience, which embraced a large number of dispensary cases of the worst class, anchylosis had not occurred in a single one of the cases which he had treated during the past four years. The members would, doubtless, recall the cases that he had previously presented, which, although completely immobilized for periods of about one year, still had complete motion of the joint. He did not believe that fixation of a joint, either diseased or healthy, resulted in anchylosis. The fact that anchylosis was not a constant result of fixation, proved this theory to be erroneous. The "ossified man," during the early stages of his disease, had been subjected to all sorts of manipulations, yet every joint became anchylosed. He believed the case of anchylosis reported in the paper was due to some affection of the nervous system, and was not the result of the immobilization. Anchylosis is determined by the character of the inflammation, its severity and duration, the parts involved, and the subsequent cicatricial contraction of the capsule of the joint, and he could not see how passive motion could prevent such destructive changes. The long traction splint, no matter how applied, will allow the foot to be elevated 35°, by tilting of the band at the pelvis. He preferred this instrument, however, to the short traction splint. Although he had employed lateral traction at first without knowing that it had been used before, he had since found several references to it in literature, showing that it had been used many years ago by Busch.

While on the subject of the use of the long traction splint, he wished to call to mind the fact that cases of hip-joint disease present great differences, and that some which run a favorable course are accompanied by much pain, while others which are associated with extensive destruction of bone have very little pain. He hoped that everyone using the long traction splint would have as fortunate an experience as had the author, but for the present he felt that he must continue to use his lateral traction splint.

Dr. John Ridlon said that, in a paper which he had written a few years ago on the subject of Fixation and Traction, he stated that as he had never met with a patient who had worn the short splint, he thought this splint could not be used much in this vicinity. He wished to take this opportunity to say that, since writing that paper, he had seen three cases which had previously worn this splint. He had been especially interested in Dr. Sayre's statement that he had secured better results with this instrument than with the long traction splint. Some years ago, he had come to the conclusion that the long traction splint was positively harmful as a walking apparatus, as it seemed to increase "the pumping action" at the joint. That it should do so, seemed reasonable when one recalled the fact that, with a traction of from five to ten pounds, and a splint weighing from six to eight pounds, the patient at each step stands upon the splint, lifting the well leg and relaxing all traction. The effect of this upon the joint can be easily imagined, when it is remembered that a child running about takes two or three thousand steps an hour. That this splint does exert a harmful influence in this way, seems to be still further confirmed by the better results which the author had obtained from the short traction splint. As many of the cases had been treated at different times by both the long and the short splints, it was difficult to say how much of the good result was to be attributed to the one or the other splint. It seemed to him that some cases of hip joint disease seemed to recover, no matter what the method of treatment adopted, or even when they were entirely untreated. We had not yet found out what was the essential vital principle in the treatment of each individual case. As an instance of this, he cited the case of a child whom he had treated most carefully for six years, and yet the result was not as good as in the case of a sister of this child who had gone through the entire period of hip disease without any surgical treatment. It was true that some of his cases which should be on crutches were walking around on the limb, because he was unable to control them, yet he was free to admit that it did not seem to have hurt them.

Dr. Halsted Myers said that the majority of cases of tubercular osteitis of the hip have the primary local focus in the neck of the femur at the junction of the epiphysis and shaft. We can recognize this condition by appropriate tests, and as at this stage there is no involvement of the cartilages of the joint, it is obviously unnecessary to immobilize the joint; yet it is *most important* that concussion and pressure should be taken from the inflamed and softened bone, and that there should be no possibility of the weight of the body being thrown on that limb. He believed that in a number of cases the disease never extends beyond this location, and is cured *in situ*. He had no pathological specimens to prove this point, nor has it been investigated as yet; he spoke from a clinical standpoint. In cases where there is erosion of the joint surfaces bearing against each other, he thought motion is injurious, as well as pressure, and is plainly indicated by the presence of reflex muscular spasm, which is a reliable guide. We always find reflex muscular spasm at the point where motion is injurious. On the other hand, immobilization of a disorganized joint, *provided pressure is also relieved*, he had never seen to cause any permanent injury to the joint. To show the importance of the relief of pressure in this connection, he stated that in order to relieve pain, he had had to apply traction to a case of hip disease which was wearing a Thomas splint, correctly shaped and applied. Recognizing the importance of this evidence, he had made repeated careful observations, but always with the same result, that traction was in this case necessary for the relief of pain.

Dr. H. W. Berg wished to protest against the feeling of nihilism which might be engendered by Dr. Ridlon's remarks. If we were able to make a purely pathological diagnosis instead of a generic one—"hip disease"—we might be able to point out in advance those cases which would do well and those which would do ill.

Dr. W. R. Townsend said that while not wishing to detract in the least from the credit due the author for securing such excellent results, he desired to point out the fact that one factor contributing to this end, was undoubtedly the very favorable surroundings of his patients. Again, the author could hardly have selected better cases had he desired to illustrate the traumatic origin of hip disease; and the fact of many of the cases reported having such an origin, affords still further reason for the excellence of his results. Bone tuberculosis and an osteitis due to traumatism may give the same clinical symptoms, but they should give different ultimate results.

Dr. Judson said that for a number of years he had kept a description of all the hip splints he had applied, and their weight had ranged from one to one-half pounds in the case of a child to little over five pounds for a large adult.

He thought that some of us were dissatisfied with the hip splint because we expect more than the nature of these cases allows. We cannot cut short hip disease as we can break up chills with quinine. We must put the part and the system in the most favorable position attainable, and then wait for the processes of natural repair. This is best done by making traction, so long as it is needed, and protecting the limb throughout the treatment from the traumatism of walking, while locomotion is freely practised. Traction and protection are the features of the American method, by which it is distinguished from the Liverpool method of portable leverage, and the London method of recumbent traction. The results obtained by Dr. Sayre are good, but not exceptional. They are within the reach of all who adhere to the plan of treatment which has been outlined.

Dr. R. H. Sayre said that the fact that one man regards a case as tubercular, and another as non-tubercular, did not change the character of the lesion, nor influence the progress of the disease.

Regarding the question of the occurrence of anchylosis, he said that he believed some cases would become anchylosed whether motion was allowed, or entirely prevented, and as an illustration of this he recalled a case of double hip joint disease in which the disease on one side was very severe, and was accompanied by extensive suppuration, while on the other side it ran a much milder course. During the progress of the disease in the latter joint, she was kept in bed or in a wire cuirass, yet notwithstanding this treatment and the apparently mild course of the disease, absolute anchylosis was the result, while in the other joint good motion was secured. Again, after the disease was apparently arrested in both joints, and both seemed to be equally stiff, passive motion gave a good joint on the side which had suppurated, but resulted in no benefit to the other side. He had seen a number of cases of disease of both hips and knees, in which the joints seemed to be perfectly fixed until passive motion was instituted. He did not approve of leaving these stiffened joints to be loosened up by the ordinary motions which the patient would make.

Dr. Phelps said he agreed with the other speakers as to the value of forcible breaking up of adhesions under ether, but he could not understand how motion of a joint during inflammation could *prevent* anchylosis. As the inflammatory material which limits the motion during inflammation is absorbed, there will be an increased motion of the joint, and, in his opinion, active motion on the part of the patient was better than passive motion. He had frequently produced by passive motion a return of the pain and stiffness in the joint.

Dr. Townsend could not see how anyone could believe that an osteitis due to traumatism represented the same pathological process as one due to tuberculosis, although the clinical symptoms might be identical.

The Chairman said that while all must admit that the statistics presented in the paper are not only brilliant, but exceedingly valuable, in comparing them with the statistics of those who do not resort to excision of joints, allowance must be made for those joints which had gone on to excision. This would also affect the mortality. One point which was very strongly brought out in the paper, was the positive, decided, and immediate relief of pain obtained in the majority of cases from traction properly applied. In hip joint disease, it is fair to infer, as is also evident from the results obtained, that if the pain is relieved, the treament is beneficial to the joint. He believed in immobilization in the acute stage, so far as it could be produced by traction, but he did not believe it was necessary to go up to the axilla and immobilize the spinal column. Sometimes traction must be supplemented by recumbency, and sometimes by the use of crutches ; these were all the necessary elements for the proper management of those cases which can be successfully treated by mechanical means. His own experience had led him to think that by far the most efficient method of applying traction was by means of the long traction splint.

Dr. SAYRE, in closing the discussion, said that the statistics presented were only those which had been fully completed, and they represented forty years of work. He thought Dr. Phelps had misunderstood him about the question of motion at the joint. He had always advocated, repeatedly and persistently, rest of an inflamed joint, but he *permitted* such motion as the patient would himself make. He did not consider that any motion which would not cause pain was injurious. He applied sufficient traction to prevent pressure on the joint, and it was all-important that this traction should be made in the proper direction. He did not approve of an unyielding strap, which, in the splint used by Dr. Taylor and Dr. Shaffer, is attached to the pelvic band and the shaft of the splint ; in his opinion, it should be made of elastic webbing.

As regards the etiology of his cases, he did not pretend to say whether or not the processes were tubercular or non-tubercular. At the time he began his investigations, everything was called "scrofula," and medical men believed that tubercle was always found in the lungs before it was deposited in other parts of the body. Having learned from autopsies on some cases of hip-joint disease that there were no tubercles in the lungs, he began to doubt the tubercular nature of this disease, and he was led to look upon it as a chronic inflammation resulting from a greater or less degree of traumatism. Now that the presence of tubercle bacilli furnished a definite basis for a diagnosis, he was trying to learn something about the occurrence of tubercle in these cases. Clinical experience had taught him, however, that whether these cases were tubercular or not, fresh air, good food, and freedom from pain were the essentials for a cure.

Referring to the occurrence of anchylosis, he said that one single case of absolute, firm anchylosis of all the joints in the body was worth more to him than any number of experiments on dogs. In the case which he had reported in his paper there was no fever, no evidence of any nervous derangement, in fact, there was no constitutional disturbance. To apply a splint without traction is wrong ; nothing makes better immobilization than plaster of Paris, and it is much more comfortable than the Thomas brace, yet it is insufficient, without traction, to overcome the reflex muscular contraction, and to relieve pain. The treatment which he advocated was the best possible one, no matter what the etiology of the disease.

Dr. John Ridlon exhibited a convenient pocket-knife, with blades especially designed to facilitate the removal of plaster of Paris bandages.

